# A novel nonsense mutation in *WNK1/HSN2* associated with sensory neuropathy and limb destruction in four siblings of a large Iranian pedigree

**DOI:** 10.1186/s12883-018-1201-6

**Published:** 2018-11-29

**Authors:** Behrouz Rahmani, Fatemeh Fekrmandi, Keivan Ahadi, Tannaz Ahadi, Afagh Alavi, Abolhassan Ahmadiani, Sareh Asadi

**Affiliations:** 1 Section of Physiology, Department of Basic Sciences, Faculty of Veterinary Medicine, University of Tehran, Tehran, Iran; 2 Neuroscience Research Center, Shahid Beheshti University of Medical Sciences, Tehran, Iran; 3 Department of Radiation Oncology, University Health Network, Princess Margaret Cancer Centre, Toronto, Canada; 4 Department of Orthopaedic Surgery, Milad Hospital, Tehran, Iran; 5 Neuromusculoskeletal Research Centre, Department of Physical Medicine and Rehabilitation, Iran University of Medical Sciences, Tehran, Iran; 6 Genetics Research Center, University of Social Welfare and Rehabilitation Sciences, Tehran, Iran

**Keywords:** Hereditary sensory and autonomic neuropathies, HSAN2, Nonsense mutation, Whole exome sequencing, *WNK1* gene

## Abstract

**Background:**

Hereditary sensory and autonomic neuropathy type 2 (HSAN2) is an autosomal recessive disorder with predominant sensory dysfunction and severe complications such as limb destruction. There are different subtypes of HSAN2, including HSAN2A, which is caused by mutations in *WNK1/HSN2* gene.

**Methods:**

An Iranian family with four siblings and autosomal recessive inheritance pattern whom initially diagnosed with HSAN2 underwent whole exome sequencing (WES) followed by segregation analysis.

**Results:**

According to the filtering criteria of the WES data, a novel candidate variation, c.3718C > A in *WNK1/HSN2* gene that causes p.Tyr1025* was identified. This variation results in a truncated protein with 1025 amino acids instead of the wild-type product with 2645 amino acids. Sanger sequencing revealed that the mutation segregates with disease status in the pedigree.

**Conclusions:**

The identified novel nonsense mutation in *WNK1/HSN2* in an Iranian HSAN2 pedigree presents allelic heterogeneity of this gene in different populations. The result of current study expands the spectrum of mutations of the *HSN2* gene as the genetic background of HSAN2A as well as further supports the hypothesis that *HSN2* is a causative gene for HSAN2A. However, it seems that more research is required to determine the exact effects of this product in the nervous system.

## Background

Hereditary sensory and autonomic neuropathies (HSANs) are inherited group of neurodegenerative disorders of the peripheral nervous system associated with sensory dysfunction [1]. HSAN is characterized by the multimodal loss of sensation with or without presentation of autonomic disturbances [2]. In this group of neuropathies, motor neurons are relatively or entirely spared [3]. Based on clinical features and pattern of inheritance, HSAN has been categorized into seven types with additional entities related to mutations in different genes [4].

HSAN type 2 (HSAN2) was first described in 1973 by Ohta M. et al. [5], but its genetic etiology was initially demonstrated from Canadian patients in 2004, and causative mutation was reported in an intron within the *WNK1* gene, referred to as *HSN2* [6]. HSAN2 occurs sporadically or with autosomal recessive inheritance pattern, which is usually diagnosed during the first two decades of life [7]. Predominantly, HSAN2 patients present sensory deficit in distal lower limbs more severely than the upper ones with possible motor involvement and variable autonomic disturbances. With the passing of time, ulcero-mutilating complications will be revealed and, the disease can become more complicated by osteomyelitis and painless fractures [2, 8]. This disorder doesn’t have a sex preference or particular ethnic distribution [9].

The recommended approach taken to the diagnosis of HSAN2 is based on detailed family history, clinical and paraclinical findings comprising neurological examinations in order to determine the extent of sensory loss and involvement of autonomic and motor nervous system, electrophysiology, histopathological evaluation of sural nerve and molecular genetic testing of candidate genes [8]. Nowadays, the genetic methods such as next-generation sequencing (NGS) based methods, are good opportunities to reduce the requirement of invasive diagnostic tests such as histological evaluation [10].

HSAN type 2 is classified into four subtypes including HSAN2A, HSAN2B, HSAN2C and HSAN2D that respectively are ascribed to genes *HSN2*, *FAM134B*, *KIF1A* and *SCN9A* in which several mutations are known to develop the diseases [11–13].

*HSN2* (NM_213655) is a single-exon gene located within intron 8 of *WNK1* (WNK lysine deficient protein kinase 1). *WNK1/HSN2* appears to be expressed in satellite and Schwann cells, and sensory neurons [14]. Various *HSN2* mutations have been reported from patients with different ethnicity causing HSAN2 [1, 6, 14, 15]. Herein, we report a novel nonsense mutation in *WNK1/HSN2* associated with the clinical presentations of HSAN2 in four siblings of an Iranian family.

## Subjects and methods

**Fig. 1:**
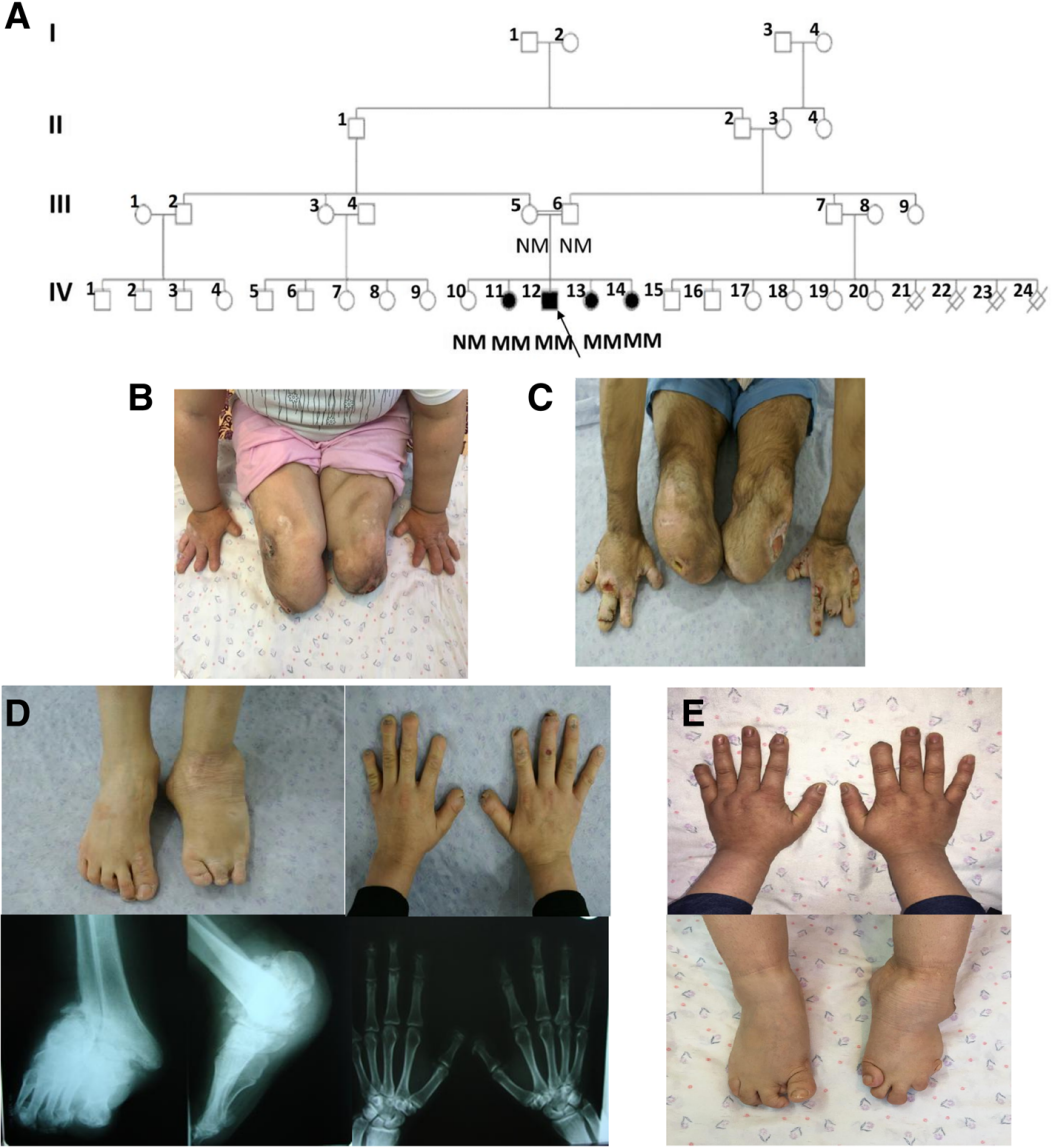
The pedigree and clinical appearance of the studied family. **a** Iranian HSAN2A pedigree with a mutation in *WNK1/HSN2* gene. Genotypes of studied individuals are presented. Filled circles and squares, affected individuals; unfilled circles and squares, unaffected members; Arrow shows proband. M, mutated allele; N, normal allele; B, C. Chronic ulcers as well as the amputated and mutilated sites on upper and lower extremities of the HSAN2-IV: 11 (**b**) and HSAN2-IV: 12 (**c**). D, E. Fingers deformity and Charcot joint in the left foot of the patients HSAN2-IV: 13 (**d**) and HSAN2-IV: 14 (**e**)

An HSAN2 pedigree with four affected and one unaffected sibling was recruited (Fig. 1a). The HSAN2 diagnosis was made based on clinical criteria, the results of electrophysiological evaluations, audiometry and tympanometry tests and laboratory assessments. The clinical features of the patients were recorded and summarized in Table 1.

**Table 1:** Clinical features and results of genetic analysis of four affected individuals with *WNK1/HSN2* mutation

	HSAN2- IV:11	HSAN2-IV:12	HSAN2-IV:13	HSAN2-IV:14
Age at onset (y)	6 months	2	7	10
Present age (y)	36	32	30	27
Sex	Female	Male	Female	Female
First symptoms	Consecutive limb ulcerations and infections	Consecutive limb ulcerations and infections	Consecutive limb ulcerations and infections	Consecutive limb ulcerations and infections
Self mutilation	Yes	Yes	Yes	Yes
Amputation	Bilateral transtibial	Bilateral transtibial	Left 3rd and 5th toes	Left 5th toe
Sensory involvement of distal extremities				
Deep tendon reflexes	Reduced	Reduced	Reduced	Reduced
Pain perception	Absent	Absent	Severely Reduced	Severely Reduced
Touch perception	Absent	Absent	Severely Reduced	Severely Reduced
Temperature sensation	Absent	Absent	Severely Reduced	Severely Reduced
Vibration sensation	Reduced	Reduced	Reduced	Reduced
Position sensation	Reduced	Reduced	Reduced	Reduced
Pressure sensation	Reduced	Reduced	Reduced	Reduced
Motor dysfunction	No	No	No	No
Autonomic involvement				
Gastroesophageal reflux	No	No	No	No
Constipation	Yes	No	No	No
Orthostatic hypotension	No	No	No	No
Episodic hypertension	No	No	No	No
Recurrent fever episodes	No	No	No	No
Hearing impairment	No	No	No	No
Skin hyperkeratosis	Yes	Yes	Yes	Yes
Lack of fungiform papillae	No	No	No	No
Mental development	Normal	Normal	Normal	Normal
Genotype	MM	MM	MM	MM

*y* year, *M* Mutant allele

### Genetics evaluations

According to the disease inheritance pattern, patients’ clinical history, and presentations, which complied with HSAN2, we initiated the molecular genetics evaluations with focused effort on this disease. Genomic DNA of all family members was extracted from blood samples using salting out protocol. The DNA fragments of genes *WNK1*, *KIF1A*, *FAM134B*, and *SCN9A* in which the prior mutations have been reported to be responsible for HSAN types 2 were respectively amplified by polymerase chain reaction (PCR). The amplicons were sequenced by Sanger method (Applied Biosystems, Foster City, CA). Afterwards, the extracted data were compared with relevant human reference sequences accessible in NCBI.

### Whole exome sequencing

**Fig. 2:**
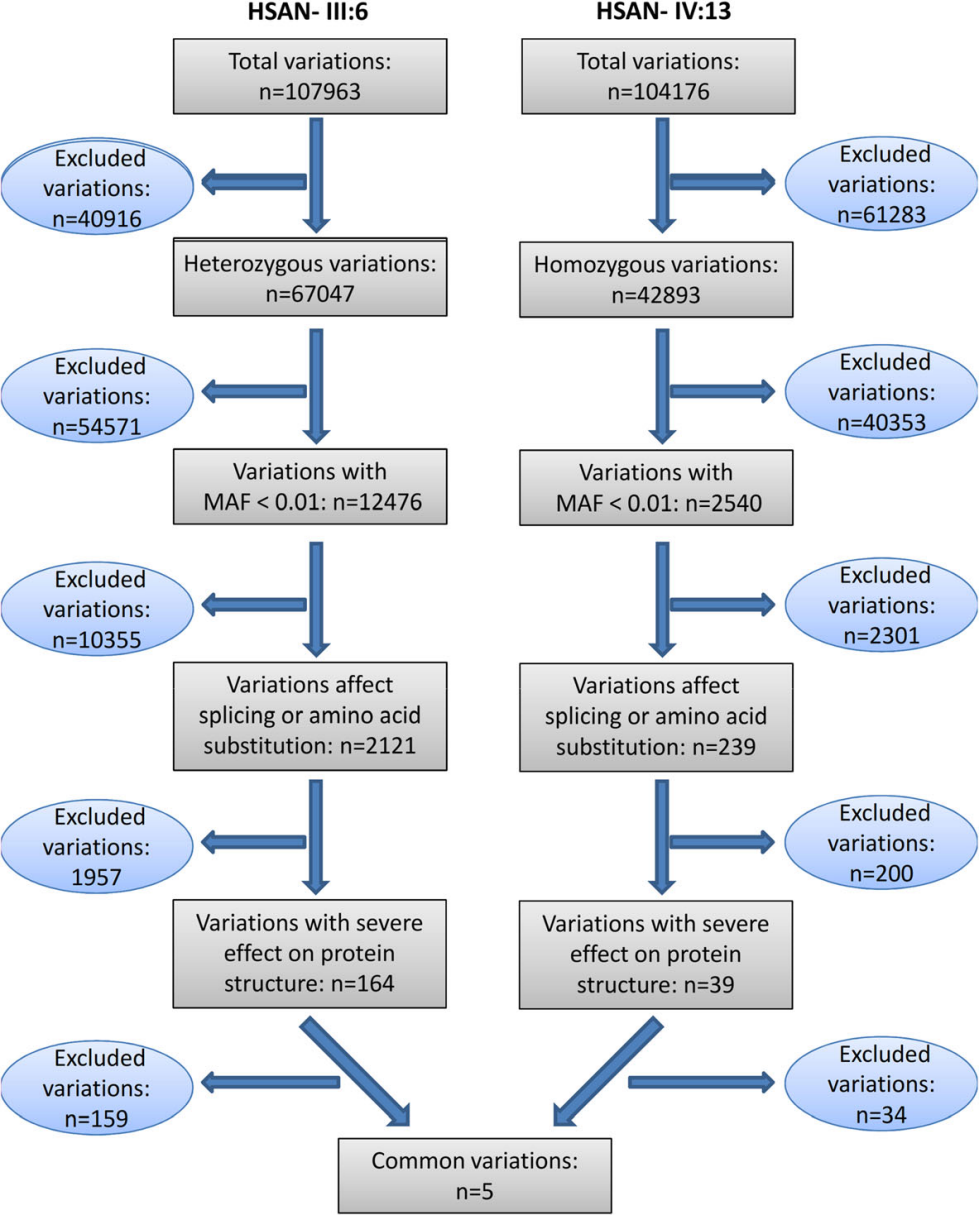
Flow diagram of WES data filtering process

Whole exome sequencing (WES), Illumina HiSeq®2000 system (Illumina) was performed on the DNAs of two affected individuals HSAN- III:6 and HSAN- IV:13. Sequence alignment and variant calling were performed against human reference genome UCSC NCBI37/hg19. WANNOVAR (http://wannovar.wglab.org/) and ENSEMBL (http://asia.ensembl.org) are used to annotate the functional consequences of genetic variations. Based on the autosomal recessive inheritance of the disease and consanguinity of the parents, all heterozygous variations in father (HSAN- III:6) and homozygous variations in the proband (HSAN- IV:13) were selected. Subsequently, with the assumption that HSAN2 is a rare disease, SNPs with a reported minimal allele frequency of > 0.01 in the dbSNP database (http://www.ncbi.nlm.nih.gov/), the 1000 Genome project (http://www.internationalgenome.org/), or the NHLBI Exome Sequencing Project (http://evs.gs.washington.edu/EVS/), GnomAD (http://gnomad.broadinstitute.org/) and Iranome (http://www.iranome.com/) were removed. In the next step, variants did not affect splicing, or amino acid change (e.g. synonymous, 3UTR, 5UTR variations) were filtered out which was followed by the removal of variations with mild or moderated effect (Fig. 2).

### Mutation screening

Using specific primers (fwd: 5’-ATTTCCCAGCGGCGTAAG-3′ and rev: 5’-CATTGAGACGTCAGAGCCA-3′), 984 bp of the *HSN2* exon of *WNK1* containing the candidate variation -c.3718C > A- was amplified, subsequently genotyped by Sanger sequencing (Applied Biosystems, Foster City, CA). *WNK1* reference sequences used were NC_000012.12, NM_001184985, and NP_ 001171914.

## Results

### Clinical evaluations

#### HSAN2- iv: 11

A 36-year-old girl with below knee-amputated limbs and the mutilation of all distal phalanges of her upper extremities. She reported consecutive ulcers on the lower extremities from the age of six months old. Following deep ulcer infections and osteomyelitis occurrence, below knee amputations, were preceded on the left at age nine years old and on the right 13 years later (Fig. 1b).

#### HSAN2-iv: 12

A 32-year-old male subject, whose limb ulcerations and infections were initiated in the second year of his life. Bilateral transtibial amputations were done when he was 12 years old. The finger mutilation of his hands is shown in Fig. 1c.

#### HSAN2-IV: 13 and HSAN2-IV: 14

The third and fourth daughters of the family are aged 30 and 27 years old. They presented the first symptoms of consecutive limb ulcerations and infections, in seven and 10 years old, respectively. Over the time, the finger mutilation and deformity of upper and lower limbs occurred with prominent Charcot neuropathic arthropathies in their left feet (Fig. 1d and e). After profound infections around the ages between 10 and 15, the left third toe of HSAN2-IV: 13, the right second toe of HSAN2-IV: 14, and in both cases, the fifth finger of left lower extremities were amputated.

In all patients, there is a history of non-progressive symmetrical reduced multimodal sensory function in distal areas of upper and lower extremities. In the clinical examinations, pain, temperature, vibration, position, pressure, fine and crude touch perception had decreased in distal half of lower limbs in cases IV:11 and IV:12 as well as in distal one-third of lower extremities in cases IV:13 and IV:14, with the distal end of upper limbs in all patients. The sensory impairment was more profound in pain, touch and temperature sensation with more severity in cases IV: 11 and IV: 12. Moreover, deep tendon reflexes were diminished in all patients. The clinical examinations of motor neurons, autonomic systems and cranial nerves, had no abnormal findings. All patients were in normal cognitive status. They had normal tickling sensation and sexual activity. Skin exam showed hyperkeratosis in the limbs and the lingual fungiform papillae were observed in the oral examination. Besides, there was history of neither hypertension nor recurrent fever episodes in the family (Table 1).

Electrophysiological evaluations including sensory and motor conduction studies, F wave and H reflex analysis, electromyography and the sympathetic skin response test which have been done in cases IV: 13, 14 revealed symmetric peripheral sensory neuropathy, axonal type, with the impaired skin sympathetic response. The audiometry and tympanometry tests confirmed normal hearing. Laboratory assessments of fasting blood sugar (FBS), liver function tests (LFT), electrolytes, blood urea nitrogen, creatinine, and urine analysis were normal in all patients.

### Genetics evaluations

**Fig. 3:**
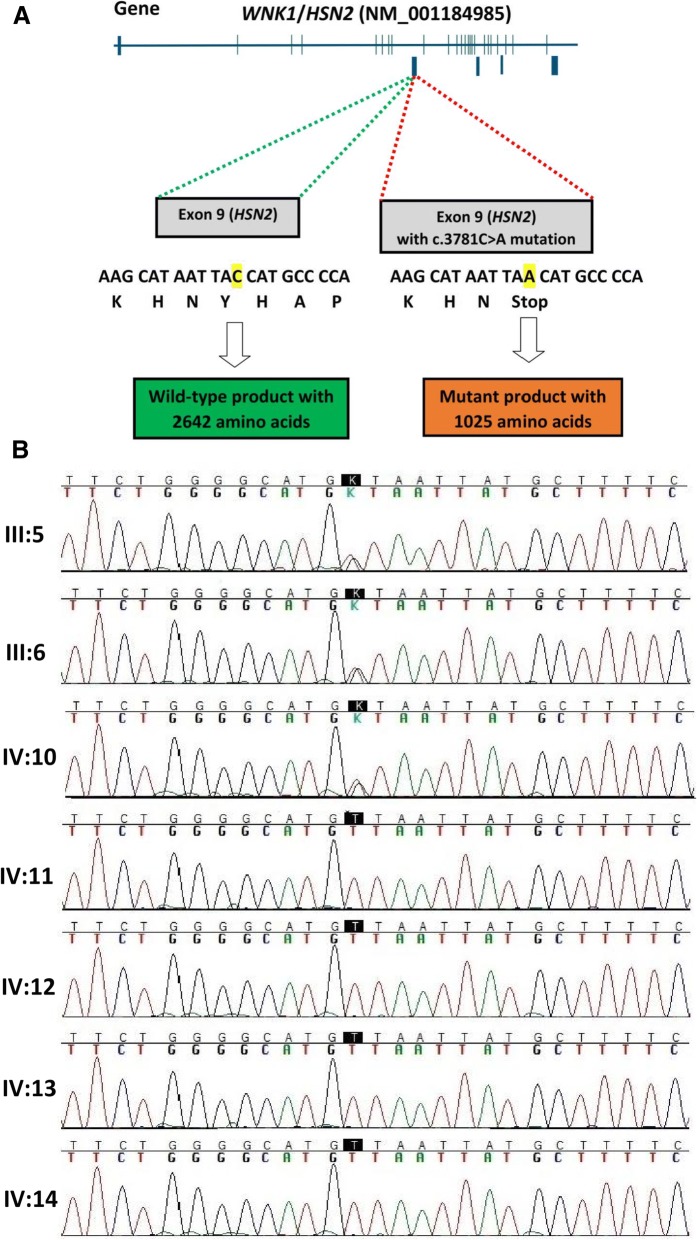
**a** The schematic diagram of *WNK/HSN2* gene. The identified nonsense mutation c.3718C > A in HSN2 exon of WNK1 transcript alters the tyrosine codon TAC to the stop codon TAA which leads to a truncated protein. **b** Direct sequencing of the HSN2 amplicon containing the mutation in all the members of the affected family. Healthy subjects are in heterozygous status (III:5, III:6, and IV:10) but affected members (IV:11, IV:12, IV:13, and IV:14) are homozygous for this mutation

Sanger sequencing of PCR products containing previously mutations in *WNK1*, *KIF1A*, *FAM134B*, and *SCN9A* genes showed wild type alleles; hence, we decided to perform the WES to have a better outline of genetic variations. According to the filtering criteria of the WES data a novel candidate variation, c.3718C > A in *WNK1/HSN2* gene that causes p.Tyr1025* was identified. This variation results in a truncated protein with 1025 amino acids instead of the wild-type product with 2645 amino acids; p.Tyr1025* (Fig. 3a) and segregated with disease status in the pedigree. All affected members were in the homozygous state while unaffected parents and sibling were in the heterozygous state (Fig. 3b). Also, c.3718C > A was not observed in the Iranome (http://www.iranome.ir/) and other public databases. Given that, variations in this gene have been reported as a cause of HSAN2A, so, it was assessed as the likely cause of the neurologic disorder in this family.

## Discussion

With-no-lysine (K) kinase 1 (WNK1), lysine deficient protein kinase 1, a gene located on 12p13.33, consists of 28 coding exons and encodes multiple isoforms of serine/threonine-protein kinase WNK1. WNK1 proteins regulate the activity of other proteins by attaching the phosphate group to specific positions. Due to the protein kinase properties, WNK1 isoforms can control the fluxes of sodium and chloride ions and have been considered as one of the primary regulators of blood pressure. Accordingly, several mutations resulted in *WNK1* over-expression have been reported as the causative mutations of an autosomal dominant disorder, pseudohypoaldosteronism type II, which is characterized by hypertension, hyperkalemia, and renal tubular acidosis [8, 16]. In summary, it has been suggested that WNK1 proteins in cooperation with the other WNK kinases and with mediation of kinases SPAK and OSR1 have role in renal epithelial transport, maintaining cell volume homeostasis, GABA signaling, immune function and cell migration through the effect on cation-Cl^−^ co-transporters [17].

HSN2 is an alternatively spliced exon in WNK1 providing the nervous system specific isoform of WNK1 transcript, namely *WNK1/HSN2* [14]. It has been hypothesized that *WNK1/HSN2* product involves the control of neuronal ion transportation and affect membrane excitability. Furthermore, several lines of studies have been shown this protein regulates neurite extension via its effect on Nogo signaling during neuronal differentiation [18, 19]. To date, 20 homozygous mutations in *WNK1/HSN2* have been reported and almost all are in the *HSN2* exon leading to truncating and loss-of-function [15] and WNK1/HSN2 protein leads to HSAN2A disease [6, 20–23]. Analyzing the results of genetics evaluations including Sanger sequencing of previously reported mutations as well as exome sequencing showed that none of the previously reported variations for HSAN2 were present in this family, but a novel nonsense mutation c.3718C > A in *HSN2* exon of *WNK1* transcript was found that resulted in alteration of the tyrosine codon TAC to the stop codon TAA and a truncated protein with 1025 amino acids. This result has been confirmed by subsequent segregation analysis and introduced c.3718C > A as a novel mutation for HSAN2A.

Clinical symptoms of the prior reported HSAN2A patients characterized within first two decades of life with predominant sensory polyneuropathy manifested most profound distally in a glove-stocking distribution. Sensory neuropathy was reported in lower limbs more severely than the upper limbs, although the trunk was involving in some patients. In addition, the previously reported patients presented with decreased or absent of DTRs, limb ulceration and mutilation with or without muscle weakness and variable autonomic disturbances [6, 15, 21, 23]. Our studied patients’ clinical characteristics include reduced perception to pain, touch, sense of temperature, and vibration and position sensation in distal areas of upper and lower extremities with more severity in the lower limbs. They express reduced DTRs, limb mutilation and amputation, with unremarkable autonomic clinical examination without motor dysfunction (Table 1) which are consistent to the clinical presentations of previously reported data in patients with HSAN2A [8].

Clinical presentations, inheritance pattern, and genetic analyses in our reported patients confirmed the HSAN2A diagnosis by identification of a novel mutation. It is worth mentioning there are several reports of HSAN2 patients from Iran but none of them conducted genetic investigations [24–27]; thus the current study is the first report of HSAN2A in Iranian patients with complementary genetic analyses. Detection of this new mutation in *HSN2* presents allelic heterogeneity of this gene in different populations.

Consistent with previously reported mutations in *HSN2* exon, the novel mutation identified in the current Iranian HSAN2 pedigree also leads to a truncating loss-of-function mutation. Therefore, the result of present study expands the spectrum of mutations of the *HSN2* gene as the genetic background of HSAN2A as well as further support the hypothesis that *HSN2* is a causative gene for HSAN2A. However, more research is required to determine the exact effects of this mutation in the nervous system. Moreover, the relevance of WNK1/HSN2 transcript and its pathogenic mutations to HSAN needs more deciphering.
